# Aminoguanidine Exerts a β-Cell Function-preserving Effect in High Glucose-cultured β-Cells (INS-1)

**DOI:** 10.1155/EDR.2000.111

**Published:** 2000

**Authors:** Yuji Tajiri, Valdemar Grill

**Affiliations:** 1 aDepartment of Molecular Medicine The Endocrine and Diabetes Unit Karolinska Hospital Karolinska Institute Stockholm S-17176 Sweden; 2 Endocrine Section Department of Medicine University of Trondheim Trondheim N-7006 Norway

## Abstract

We investigated the effects of aminoguanidine (AG) on *β*-cell functions in an insulin secreting cell line (INS-1). Culture with 27 mM glucose for one week markedly decreased both insulin release and insulin content compared to culture in 0.8 mM or 3.3 mM glucose. Relative to culture at 27 mM glucose alone, the co-exposure to 1 mM AG almost doubled basal as well as glucose or 25 mM KCl-stimulated insulin release and increased insulin content by 42%. AG failed to affect release and content in cells cultured at 0.8 or 3.3 mM glucose. Preproinsulin mRNA content in 27 mM glucose-cultured cells was 52% suppressed compared to 0.8 mM glucose-cultured cells, and AG treatment partially counteracted this decline. Advanced glycosylation end product (AGE)-associated fluorescence (370 nm excitation and 440 nm emission) of cells′ extracts did not differ between 27 mM and 0.8 mM glucose-cultured cells after 1 week of culture and fluorescence was unaffected by AG. Accumulation of nitrite into culture media was markedly increased from 27 mM glucose-cultured cells, and this accumulation was 33% suppressed by AG. In conclusion, AG partially protects against glucotoxic effects in INS-1 cells. These beneficial effects may involve a decrease in early glycation products and/or nitric oxide synthase (NOS) activity. The effects which were obtained after one week of high glucose exposure may supplement AGE-associated effects seen after chronically elevated glucose.


*Int. Jnl. Experimental Diab. Res.*, Vol. 1, pp. 111–119
Reprints available directly from the publisher
Photocopying permitted by license only

© 2000 OPA (Overseas Publishers Association) N.V.
Published by license under
the Harwood Academic Publishers imprint,
part of The Gordon and Breach Publishing Group.
Printed in Malaysia.

### Aminoguanidine Exerts a β-cell Function-preserving Effect in High Glucose-cultured β-cells (INS-1)

YUJI TAJIRI[a] and VALDEMAR GRILL[a,b,*]

[a]*Department of Molecular Medicine, The Endocrine and Diabetes Unit, Karolinska Hospital, Karolinska Institute, S-17176 Stockholm, Sweden;* [b]*Endocrine Section, Department of Medicine, University of Trondheim, N-7006 Trondheim, Norway*

*(Received in final form 6 February 2000)*

**We investigated the effects of aminoguanidine (AG) on β-cell functions in an insulin secreting cell line (INS-1). Culture with 27 mM glucose for one week markedly decreased both insulin release and insulin content compared to culture in 0.8 mM or 3.3 mM glucose. Relative to culture at 27 mM glucose alone, the co-exposure to 1 mM AG almost doubled basal as well as glucose or 25 mM KCl-stimulated insulin release and increased insulin content by 42%. AG failed to affect release and content in cells cultured at 0.8 or 3.3 mM glucose. Preproinsulin mRNA content in 27 mM glucose-cultured cells was 52% suppressed compared to 0.8 mM glucose-cultured cells, and AG treatment partially counteracted this decline. Advanced glycosylation end product (AGE)-associated fluorescence (370 nm excitation and 440 nm emission) of cells' extracts did not differ between 27 mM and 0.8 mM glucose-cultured cells after 1 week of culture and fluorescence was unaffected by AG. Accumulation of nitrite into culture media was markedly increased from 27 mM glucose-cultured cells, and this accumulation was 33% suppressed by AG. In conclusion, AG partially protects against glucotoxic effects in INS-1 cells. These beneficial effects may involve a decrease in early glycation products and/or nitric oxide synthase (NOS) activity. The effects which were obtained after one week of high glucose exposure may supplement AGE-associated effects seen after chronically elevated glucose.**

*Keywords:* Aminoguanidine, β-cell glucotoxicity, NO synthesis, advanced glycosylation end products, INS-1 cells

### INTRODUCTION

Aminoguanidine (AG) is a nucleophilic hydrazine compound which potently inhibits AGE formation.[1,2] Beneficial effects of AG on diabetic complications have been reported *in vivo* and *in vitro* [2] and are associated with inhibitory effects on AGE formation.[3,4] These effects of AG are considered strong evidence for an important role of AGEs in diabetic complications.

In our own experiments we have used AG as a probe to test for the possible influence of AGE formation in hyperglycemia-induced desensitization of pancreatic β-cells. In these experiments, pancreatic β-cells were cultured for a period of 6 weeks at high glucose with or without AG. Under these conditions AG suppressed AGE formation in rat pancreatic islet and concomitantly enhanced β-cell functions such as insulin release and insulin biosynthesis.[5] These effects

*Corresponding author. Tel.: +47-73868386, Fax: +47-73867546, e-mail: valdemar.grill@medisin.ntnu.no were time dependent, since they were not observed after 1 but only after 6 weeks of exposure to high glucose and AG. This time dependence is in accord with previous short term studies in which exposure to AG leads to no effect [6] or inhibition [7] of insulin secretion.

AG has also been reported to exert effects related to the inhibition of nitric oxide synthase activity [8, 9] as well as the inhibition of free radical formation from early glycation products. [10, 11] It seemed possible that AG could exert these types of effects on $\beta$-cells which are more metabolically active and dividing than $\beta$-cells in islets of adult animals. In the present study we have therefore tested for effects of AG on insulin secretion and biosynthesis in an insulinoma cell line (INS-1) during one week culture.

### MATERIALS AND METHODS

### Materials

Aminoguanidine bicarbonate, trichloroacetic acid (TCA) and phosphate buffer saline (PBS) were purchased from Sigma Chemical Co. (St. Louis, MO). Dextran T 70 were from Pharmacia (Uppsala, Sweden). RNase-A and RNase-T1 were from Boehringer Mannheim (Mannheim, Germany). [Methyl-$^3$H]thymidine (5 µ Ci/mmol) was from Amersham International (UK). $^{35}$S-UTP (1082 Ci/mmol) was from DuPont NEN (Boston, MA). Riboprobe Gemini II Core System, pGEM-3Zf (+), Herring Sperm DNA, RQ1 DNase I and restriction enzymes (EcoRI and XbaI) were from Promega Biotec (Madison, WI). GF/C filters were from Whatman International Ltd. (Maidstone, UK). RPMI-1640 medium, penicillin, streptomycin and fetal calf serum were from Gibco (Grand Island, NY). 24 multi-well tissue culture dishes were from Nunc (Denmark).

### Cell Culture

INS-1 cells were kindly provided by Dr. Claes Wollheim (Geneva, Switzerland). Cells were grown in monolayer cultures as described previously [12] in RPMI-1640 medium containing 11 mM glucose supplemented with 10 mM HEPES, 10% heat-inactivated fetal calf serum, 2 mM L-glutamine, 1 mM sodium pyruvate, 50 µM $\beta$-mercaptoethanol, 100 IU/ml penicillin and 100 µg/ml streptomycin at 37°C in a humidified (5% $CO_2$, 95% air) atmosphere. A passage number of around 20 were used for the present experiments. Cells were seeded in 24 multi-well tissue culture dish ($1.0 \times 10^5$ cells in 1 ml of medium per well, 2 cm of diameter) and cultured for 1 week in RPMI-1640 containing 0.8, 3.3 or 27 mM glucose and with or without AG (final concentration; 1 mM). Before being added to the culture media, AG was dissolved in redistilled water at acidic conditions (pH 3.0) and brought to the same pH as the culture media as described previously. [6] The culture medium was changed every second day. The following protocols and measurements were carried out after the culture period.

### Insulin Release

Batch-type incubations were carried out as previously described.[12] Briefly, the cells in each group were washed in KRB medium [13] with the following composition: $Na^+$ 143 mM, $K^+$ 5.8 mM, $Ca^{2+}$ 1.5 mM, $Mg^{2+}$ 1.2 mM, $Cl^-$ 124.1 mM, $PO_4^{3-}$ 1.2 mM, $SO_4^{2-}$ 1.2 mM, $CO_3^{2-}$ 25 mM, pH 7.4 supplemented with 10 mM HEPES, 0.1% BSA (fraction IV, Sigma Chemical Co.). They were then preincubated in the same buffer at 37°C for 30 min in a humidified (5% $CO_2$, 95% air) atmosphere. Finally, cells were incubated in triplicate at 37°C for an additional 30 min in the absence or presence of test agents (27 mM glucose or 25 mM KCl). Aliquots of the incubation media were then removed for assay of insulin concentrations. Insulin content was measured in those cells which, during final incubations, had been incubated in glucose-free media. Culture media of the last 48 h of culture were retrieved for assay of insulin concentrations.

### Cell Proliferation

Cell proliferation was estimated by [$^3$H]thymidine incorporation basically as previously described.[14,15] During the last 24 h of culture period, 1 µCi/ml of [methyl-$^3$H]thymidine was present in culture media. At the end of the labelling period, triplicate wells of cells in each group were harvested by trypsinization, washed in PBS and sonicated in 250 µl of redistilled water (10 × 2 s, model B-12 sonifier, setting 4; Branson Ultrasonics Corp., Danbury, CT). 50 µl of the sonicate was used for the assay of protein content (Bio Rad Laboratories, Richmond, CA and the BSA standard). The remainder was precipitated in ice-cold 10% TCA for DNA measurements. The precipitate was washed twice in TCA, dissolved in 50 µl of Soluene and the radioactivity incorporated was counted in a scintillation counter.

### AGE-associated Fluorescence

Triplicate wells of cells in each group were harvested by trypsinization, washed three times in PBS, and then sonicated in 300 µl of redistilled water (10 × 2 s, model B-12 sonifier, setting 4; Branson Ultrasonics Corp., Danbury, CT). The sonicates were centrifuged at 10,000 g for 10 minutes at 4°C. Fluorescence in the supernatants was measured, using excitation at 370 nm + emission at 440 nm[16] by SPEX-1681 0.22 m spectrometer (SPEX industries, Inc. Edison, N.J.). 50 µl of the sonicate was used for assay of protein contents.

### Preproinsulin mRNA

Triplicate wells of cells in each group were harvested by trypsinization and total RNA was prepared as described by Chomczynski *et al.*[17] The quantitative analysis of preproinsulin mRNA was achieved by a solution hybridization assay using a RNA probe radiolabeled with $^{35}$S-UTP.[18] An *in vitro* synthesized 58 bp oligonucleotide corresponding to the last part of exon 3 of the rat preproinsulin II gene and flanked by BamHI and KpnI restriction sites was inserted to pGEM-3Zf(+). The resulting vector, prINS2, was linearized by EcoRI and transcribed *in vitro* with SP6 RNA polymerase in the presence of 3 µmol/L $^{35}$S-UTP for synthesis of the probe. Unlabeled sense RNA was obtained by transcription with T7 RNA polymerase after linearization with XbaI. The DNA template was removed by RQ1 DNaseI and transcripts were separated from unincorporated nucleotides on Nick columns. Three serial dilutions of each RNA sample in 20 µl 0.2 × SET (1 × SET contains 1.0% sodium dodecyl sulfate, 20 mM Tris-HCl pH 7.5 and 10 mM EDTA) were mixed with 20 µl 2 × hybridization solution (20,000 cpm probe, 1.2 M NaCl, 8 mM EDTA, 1.5 mM dithiothreitol, 50% formamide and 40 mM Tris-HCl pH 7.5). After hybridization at 70°C for 18 h, the samples were treated with 40 µg RNaseA and 100 U RnaseT1 in the presence of 100 µg herring sperm DNA for 60 min at 37°C in a volume of 1 ml. Protected probe was precipitated with 100 µl 100% TCA. Precipitates were collected on glass fiber filters (GF/C) and the radioactivity was counted in a scintillation counter. Parallel hybridizations with increasing amounts of unlabeled sense RNA allowed construction of a standard curve. The amount of preproinsulin mRNA was calculated by comparison to the standard curve.

### Nitrite Concentrations

Culture media were retrieved for the last 48 h of culture. Triplicate aliquots of the culture media (80 µl) in each group were deproteinized by the addition of 20 µl of 35% sulfosalicylic acid. Samples were incubated for 15 min at 0°C and then centrifuged for 15 min at 12,000 g. 10 µl of 0.5% naphthylenediamine dihydrochloride was added to the supernatant, together with 5% sulphanilamide and 25% concentrated $H_3PO_4$. The reaction was carried out at 60°C for 1 min. Nitrite concentrations were measured as the absorbance at 546 nm in a spectrophotometer.[19]

### Insulin Assay

Insulin was measured by RIA using rat insulin as standard, monoiodinated porcine insulin as tracer and antibody raised in our laboratory against porcine insulin. Antibody-bound insulin was separated from free insulin using Dextran T70-coated charcoal.[20] For the determination of insulin contents, cells were harvested and transferred into 200 µl of acid-ethanol (0.18 M HCl in 95% ethanol). Insulin was extracted overnight at 4°C after sonication (10 × 2 s, model B-12 sonifier, setting 4; Branson Ultrasonics Corp., Danbury, CT) as previously described.[21]

### Presentation of Results

All results are expressed as mean ± SEM. Analyses between groups were carried out as appropriate by Student's *t*-test or one-way analysis of variance (ANOVA) with Student–Newman–Keuls' test. *P* value of <0.05 was considered significant.

### RESULTS

### Cell Proliferation

The incorporation of [methyl-$^3$H]thymidine into cells cultured at 0.8 mM glucose was not significantly higher than in cells cultured at 27 mM glucose ($P = 0.3$). The latter incorporation was not significantly affected by AG treatment ($P = 0.53$). Protein content in each group was comparable as well (Tab. I). Cell number cultured with 0.8 mM glucose for 1 week was $3.55 \pm 0.73 \times 10^5$/well, a value that was not significantly different from that of 27 mM glucose ($3.86 \pm 1.19 \times 10^5$/well) or 27 mM glucose with AG ($4.25 \pm 1.36 \times 10^5$/well).

### AGE-associated Fluorescence After 1 Week's Culture

The fluorescence of cells cultured at 0.8 mM glucose for 1 week ($25.4 \pm 7.9 \times 10^6$ cps/mg protein) did not differ significantly from fluorescence in 27 mM glucose-cultured cells ($22.5 \pm 0.7 \times 10^6$ cps/mg protein). Co-culture with 1 mM AG did not affect the fluorescence in 27 mM glucose-cultured cells ($21.9 \pm 2.4 \times 10^6$/mg protein, mean ± SEM of three experiments).

### Effects of Glucose Concentration and AG During Culture on Insulin Release and Content

One week of culture with 27 mM glucose markedly reduced basal as well as 27 mM glucose- or 25 mM KCl-stimulated insulin release compared to culture with 0.8 or 3.3 mM glucose (Fig. 1). Co-culture with AG did not affect insulin release from 0.8 or 3.3 mM glucose-cultured cells. Culture with AG considerably enhanced release from 27 mM glucose-cultured cells. The effect amounted to a doubling of basal and 27 mM glucose- or 25 mM KCl-stimulated secretion (Fig. 1). However, AG failed to revive an insulin response to acute stimulation with 27 mM glucose, seen after culture at 3.3 mM glucose.

TABLE I $^3$H-thymidine incorporation and protein content in INS-1 cells after 1 week's culture

| Glucose concentration (mM) | AG (mM) | Cell proliferation (cpm/µg protein/24 h) | Protein content (µg/well) |
|---|---|---|---|
| 0.8 | 0 | 3038 ± 574 | 12.5 ± 3.9 |
| 27 | 0 | 2185 ± 431 | 16.6 ± 6.2 |
| 27 | 1 | 2742 ± 692 | 13.5 ± 2.4 |

After the labelling period with 1 µCi/ml of [methyl-$^3$H]thymidine for 24 h, cells in each group were sonicated in 250 µl of redistilled water. Fifty µl of the sonicate was used for the assay of protein content. The radioactivity of the remainder was counted.
AG; aminoguanidine. Mean ± S.E.M. of 3 experiments.

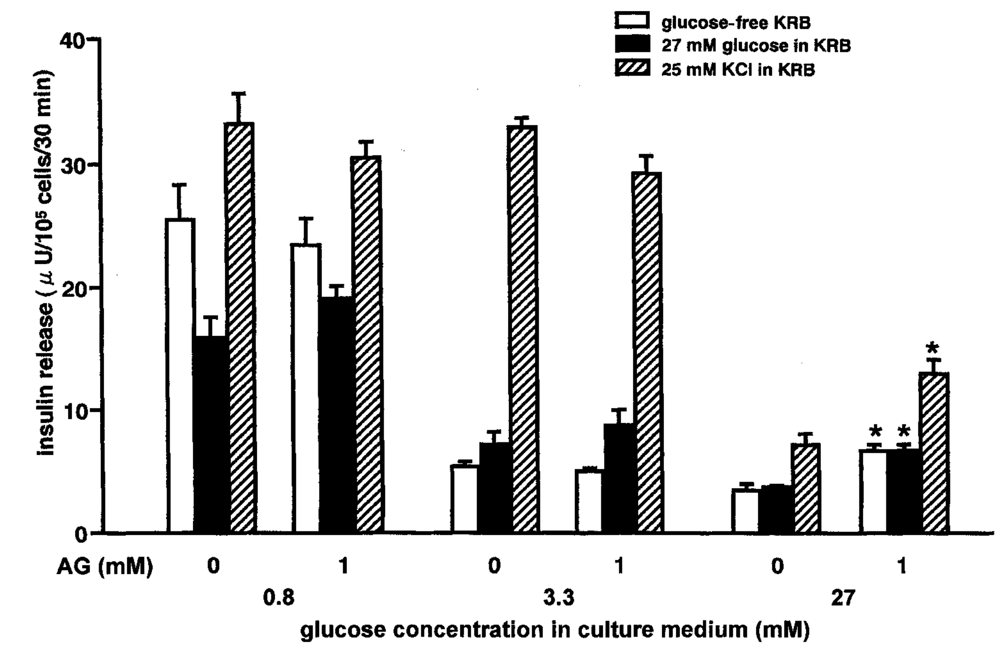

FIGURE 1 Insulin release from INS-1 cells after 1 week's culture with or without AG. After preincubation for 30 min in KRB medium without glucose the cells were incubated for 30 min in KRB in the absence or presence of test agents (27 mM glucose or 25 mM KCl). Aliquots of the incubation media were removed for assay of insulin concentrations. Mean ± SEM of five experiments. *$P < 0.05$ *vs.* AG (-).

TABLE II Insulin content and insulin accumulation into culture media in INS-1 cells after 1 week's culture

| Glucose concentration (mM) | AG (mM) | Insulin content ($\mu U/10^5$ cells) | Insulin accumulation ($\mu U/10^5$ cells/48 h) |
|---|---|---|---|
| 0.8 | 0 | 2513 ± 438 | 1078 ± 75 |
| 0.8 | 1 | 2209 ± 264 | |
| 3.3 | 0 | 1872 ± 219 | |
| 3.3 | 1 | 1886 ± 223 | |
| 27 | 0 | 241 ± 18 | 206 ± 44 |
| 27 | 1 | 343 ± 28[a] | 309 ± 38[a] |

Cells in each group which, during final incubations, had been incubated in glucose-free media were harvested and insulin content was measured after acid ethanol extraction. Culture media in each group were retrieved for the last 48 h of culture to assess insulin accumulation into culture media. AG; aminoguanidine. Mean ± S.E.M. of 3–5 experiments; [a] $P < 0.05$ *vs.* 27 mM glucose without AG.

Insulin content of 27 mM glucose-cultured cells was 90% decreased compared to cells cultured at 0.8 mM glucose and 87% decreased compared to 3.3 mM glucose. Addition of AG to cells cultured at 27 mM glucose significantly ($P < 0.05$) enhanced, but far from normalized, insulin content (by 42%). In contrast, addition of AG failed to affect insulin content in cells cultured at 0.8 or 3.3 mM glucose-cultured cells (Tab. II).

Insulin accumulation into culture media from 27 mM glucose-cultured cells was 206 ± 44 $\mu U/10^5$ cells/48 h (mean ± SEM of three experiments) and markedly decreased compared to that from 0.8 mM glucose-cultured cells (1078 ± 75 $\mu U/10^5$ cells/48 h). Co-culture with AG significantly ($P < 0.05$) enhanced insulin accumulation from 27 mM glucose-cultured cells (309 ± 38 $\mu U/10^5$ cells/48 h).

### Insulin mRNA Content

Insulin mRNA content in 27 mM glucose-cultured cells was 52% reduced compared to cells cultured at 0.8 mM glucose (Fig. 2). AG treatment significantly ($P < 0.05$) increased insulin mRNA content up to 67% of that of 0.8 mM glucose-cultured cells.

### Medium Nitrite Concentration

Medium nitrite was measured as an indicator of NO synthesis from INS-1 cells. Nitrite in culture media from 3.3 mM glucose-cultured cells was not detectable in our assay system. In contrast, 27 mM glucose induced marked accumulation of nitrite. Co-culture with AG 1 mM significantly ($P < 0.05$) suppressed nitrite accumulation by 33% (Fig. 3).

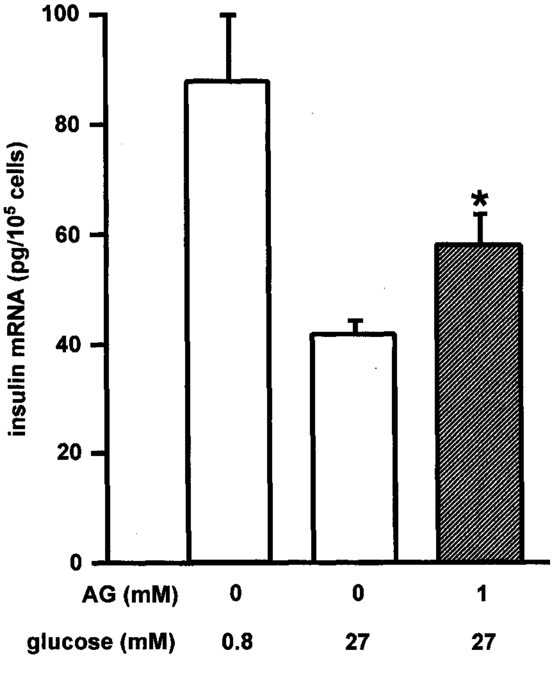

FIGURE 2 Insulin mRNA content in INS-1 cells after 1 week's culture with or without AG. Cells in each group were harvested and total RNA was prepared as described.[17] The quantitative analysis of preproinsulin mRNA was achieved by a solution hybridization assay using a RNA probe radiolabeled with $^{35}$S-UTP. Mean ± SEM of three experiments. *$P < 0.05$ *vs.* AG (-).

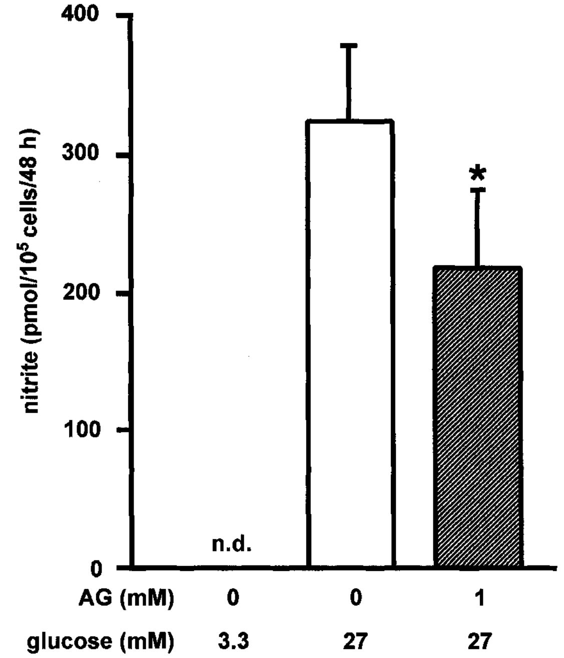

FIGURE 3 Nitrite accumulation into culture media from INS-1 cells after 1 week's culture with or without AG. Culture media were retrieved for the last 48 h of culture. Nitrite concentrations were measured as described in MATERIALS AND METHODS. n.d.; not detected. Mean ± SEM of five experiments. *$P < 0.05$ *vs.* AG (-).

### DISCUSSION

The present study demonstrates that AG exerts beneficial effects on $\beta$-cell function during short-term culture of clonal $\beta$-cells. The beneficial effects were seen specifically in high glucose-cultured cells. In those cells AG partially restored culture-induced deficiencies in insulin release, insulin content, insulin accumulation into culture media and insulin mRNA. Previous studies on a similar time scale[6,7] failed to observe beneficial AG effects. This failure may be due to the fact that AG was not tested during high glucose conditions deleterious to $\beta$-cell function.

Although the effects of AG were seen only in high glucose-cultured cells, the beneficial effects of AG were not linked to glucose-regulated modalities of $\beta$-cell function. Rather, they were generalized since, for instance, basal insulin secretion was affected together with glucose- and KCl-stimulated insulin secretion. It seems possible that beneficial effects on secretion were secondary to increased insulin content and biosynthesis.

Cell proliferation estimated by [$^3$H]-thymidine incorporation did not differ between 0.8 mM and 27 mM glucose-cultured cells in agreement with a previous study.[22] Neither was an effect of AG on cell growth apparent in the present study. Hence, the beneficial effects of AG could not be explained by effects on proliferation.

Effects of AG in biological systems were previously assigned to the inhibitory effects of the compound on AGE formation.[1,2] In a previous study[5] we found evidence for such an effect being operative also in pancreatic islets. Hence, in 6 week-cultures of rat pancreatic islets we found that high-glucose culture increased the formation of AGEs and that AG partly inhibited this formation along with beneficial effects on insulin secretion and insulin biosynthesis. However, in the present study we found neither an increase by high glucose culture of AGE-associated fluorescence, nor an effect of AG on this parameter. These findings were not surprising considering the long period necessary for the build-up of AGE products.[3,4] Indeed, in our experimental system of rapidly growing cells (doubling time 45 to 84 h, unpublished results) cells in new generations are exposed to high glucose for considerably shorter time than the one week total period of culture. From these observations we conclude that other effects of AG, not related to AGE formation, are responsible for the beneficial effects in high-glucose-cultured INS-1 cells.

Studies have demonstrated that AG exerts two non-AGE effects of potential importance, namely inhibition of nitric oxide synthase activity[8,9] and inhibition of free radical formation from glycoxidation.[10,11] Regarding the former effect we have presently demonstrated increased nitrite formation during high glucose conditions in INS-1 cells. A glucose-dependency of NO synthesis was reported also by other investigators in rat islet capillary endothelial cells[23] or HIT-T15 cells.[24] Furthermore, we find that AG inhibited significantly the accumulation of nitrite from cells cultured at high glucose. Our study does not prove a role for this effect by AG on $\beta$-cell function. However, some evidence exists that NO exerts a negative effect on insulin secretion. Thus, Panagiotidis *et al.*, reported that NO is a negative modulator of insulin release from isolated islets.[25] It thus seems possible that NO synthesis during high glucose culture is a distinct mechanism for "glucotoxicity" supplementing other mechanisms, including AGE formation in islets.[5]

It is also possible that AG exerts its beneficial effects on $\beta$-cell functions by inhibition of free radicals generated by early glycation products (Amadori products or Schiff base).[10] Matsuoka *et al.* reported that suppression of insulin gene transcription through decrease of DNA-binding activity of PDX-1 linked to ribose-induced glycation in HIT-T15 cells was prevented by both AG and an antioxidant, N-acethylcysteine, suggesting the importance of consequent increase of reactive oxygen species (ROS) for the glucotoxicity induced by glycation.[11] Similar effects have been further documented *in vitro* and *in vivo*.[26–28] Our results on insulin mRNA would agree with a transcription effect.

In cells cultured at 0.8 mM glucose the change to 27 mM glucose paradoxically inhibited insulin secretion. It is known that a low glucose concentration can induce insensitivity to glucose by mechanisms alike to those operative during *in vivo* fasting.[29] Such mechanisms may have been operative also during the present conditions.

In summary, we have demonstrated that AG partially protects against glucose- induced $\beta$-cell dysfunction in short term culture of clonal $\beta$-cells. This effect may be linked to the inhibition of free radical formation from early glycation products and/or NO synthase inhibition. These effects may supplement the beneficial effects of AG-induced inhibition of AGE formation which were evidenced in a previous study.[5]

### Acknowledgments

This work was supported by the Swedish Medical Research Council (Grant 19X-04540), the Norwegian Research Council (Grant 111282/310), the Swedish Diabetes Association, the Nordic Insulin Foundation, and Funds of the Karolinska Institute.

### References

[1] Brownlee, M., Vlassara, H., Kooney, T., Ulrich, P. and Cerami, A. (1986). Aminoguanidine prevents diabetes-induced arterial wall protein cross-linking, *Science*, **232**, 1629–1632.

[2] Brownlee, M. (1994). Glycation and diabetic complications, *Diabetes*, **43**, 836–841.

[3] Edelstein, D. and Brownlee, M. (1992). Mechanistic studies of advanced glycation end product inhibition by aminoguanidine, *Diabetes*, **41**, 26–29.

[4] Fu, M. X., Wells-Knecht, K. J., Blackledge, J. A., Lyons, T. J., Thorpe, S. R. and Baynes, J. W. (1994). Glycation, glycoxidation, and cross-linking of collagen by glucose. Kinetics, mechanism, and inhibition of late stages of the Maillard reaction, *Diabetes*, **43**, 676–683.

[5] Tajiri, Y., Möller, C. and Grill, V. (1997). Long term effects of aminoguanidine on insulin release and biosynthesis: Evidence that the formation of advanced glycosylation end products inhibits $\beta$-cell function, *Endocrinology*, **138**, 273–280.

[6] Zähner, D. and Malaisse, W. J. (1992). Effects of advanced glycation products and aminoguanidine upon insulin release, *Diab. Nutr. Metab.*, **5**, 43–46.

[7] Tasaka, Y., Nakayama, F., Matsumoto, H. and Omori, Y. (1994). Effects of aminoguanidine on insulin release from pancreatic islets, *Endocr. J.*, **41**, 309–313.

[8] Corbett, J. A., Tilton, R. G., Chang, K., Hasan, K. S., Ido, Y., Wang, J. L., Sweetland, M. A., Lancaster, J. R. Jr., Williamson, J. R. and McDaniel, M. L. (1992). Aminoguanidine, a novel inhibitor of nitric oxide formation, prevents diabetic vascular dysfunction, *Diabetes*, **41**, 552–556.

[9] Tilton, R. G., Chang, K., Hasan, K. S., Smith, S. R., Perrash, J. M., Misko, T. P., Moore, W. M., Currie, M. G., Corbett, J. A., McDaniel, M. L. and Williamson, J. R. (1993). Prevention of diabetic vascular dysfunction by guanidine-inhibition of nitric oxide synthase *vs.* advanced glycation end-product formation, *Diabetes*, **42**, 221–232.

[10] Mullarkey, C. J., Edelstein, D. and Brownlee, M. (1990). Free radical generation by early glycation products: a mechanism for accelerated atherogenesis in diabetes, *Biochem. Biophys. Res. Comm.*, **173**, 932–939.

[11] Matsuoka, T., Kajimoto, Y., Watada, H., Kaneto, H., Kishimoto, M., Umayahara, Y., Fujitani, Y., Kamada, T., Kawamori, R. and Yamasaki, Y. (1997). Glycation-dependent, reactive oxygen species-mediated suppression of the insulin gene promoter activity in HIT cells, *J. Clin. Invest.*, **99**, 144–150.

[12] Asfari, M., Janjic, D., Meda, P., Li, G., Halban, P. A. and Wollheim, C. B. (1992). Establishment of 2-mercaptoethanol-dependent differentiated insulin-secreting cell lines, *Endocrinology*, **130**, 167–178.

[13] Umbreit, W. W., Burns, R. H. and Stauffer, J. F. (1957). *Manometric Techniques*, pp. 149–150, Minneapolis, M.N., Burgess Publishing Co.

[14] Sjöholm, Å. (1991). Cytokines inhibit proliferation and insulin secretion by clonal rat insulinoma cells (RINm5F) non-synergistically and in a pertussis toxin-insensitive manner, *Immunol. Lett.*, **30**, 81–86.

[15] Janjic, D. and Asfari, M. (1992). Effects of cytokines on rat insulinoma INS-1 cells, *J. Endocrinol.*, **132**, 67–76.

[16] Monnier, V. M., Kohn, R. H. and Cerami, A. (1984). Accelerated age-related browning of human collagen in diabetes mellitus, *Proc. Natl. Acad. Sci. USA*, **81**, 583–587.

[17] Chomczynski, P. and Nicoletta, S. (1987). Single-step method of RNA isolation by acid guanidine thiocyanate-phenol-chloroform extraction, *Anal. Biochem.*, **162**, 156–159.

[18] Möller, C., Arner, P., Sonnenfeld, T. and Norstedt, G. (1991). Quantitative comparison of insulin-like growth factor mRNA levels in human and rat tissues analysed by solution hybridization assay, *J. Mol. Endocrinol.*, **7**, 213–222.

[19] Welsh, N., Eizirik, D. L., Bendtzen, K. and Sandler, S. (1991). Interleukin-1 $\beta$-induced nitric oxide production in isolated rat pancreatic islets requires gene transcription and may lead to inhibition of the Krebs cycle enzyme aconitase, *Endocrinology*, **129**, 3167–3173.

[20] Herbert, V., Lau, K. S., Gottlieb, C. W. and Bleicher, S. J. (1965). Coated charcoal immunoassay of insulin, *J. Clin. Endocrinol. Metab.*, **25**, 1375–1384.

[21] Grill, V., Rundfeldt, M. and Efendic, S. (1981). Previous exposure to glucose enhances somatostatin secretion from the isolated perfused rat pancreas, *Diabetologia*, **30**, 495–500.

[22] Robertson, R. P., Zhang, H.-J., Pyzdrowski, K. L. and Walseth, T. F. (1992). Preservation of insulin mRNA levels and insulin secretion in HIT cells by avoidance of chronic exposure to high glucose concentrations, *J. Clin. Invest.*, **90**, 320–325.

[23] Suschek, C., Fehsel, K., Kröncke, K.-D., Sommer, A. and Kolb-Bachofen, V. (1994). Primary culture of rat islet capillary endothelial cells: Constitutive and cytokine-inducible macrophagelike nitric oxide synthase are expressed and activities regulated by glucose concentration, *Am. J. Pathol.*, **145**, 685–695.

[24] Schmidt, H. H. H. W., Warner, T. D., Ishii, K., Sheng, H. and Murad, F. (1992). Insulin secretion from pancreatic $\beta$-cells caused by L-arginine-derived nitrogen oxides, *Science*, **255**, 721–723.

[25] Panagiotidis, G., Akesson, B., Rydell, E. L. and Lundquist, I. (1995). Influence of nitric oxide synthase inhibition, nitric oxide and hydroperoxide on insulin release induced by various secretagogues, *Br. J. Pharm.*, **114**, 289–296.

[26] Tanaka, Y., Gleason, C. E., Tran, P. O., Harmon, J. S. and Robertson, R. P. (1999). Prevention of glucose toxicity in HIT-T15 cells and Zucker diabetic fatty rats by antioxidants, *Proc. Natl. Acad. Sci. USA*, **96**, 10857–10862.

[27] Kaneto, H., Kajimoto, Y., Miyagawa, J., Matsuoka, T., Fujitani, Y., Umayahara, Y., Hanafusa, T., Matsuzawa, Y., Yamasaki, Y. and Hori, M. (1999). Beneficial effects of antioxidants in diabetes: possible protection of pancreatic β-cells against glucose toxicity, *Diabetes*, **48**, 2398–2406.

[28] Kajimoto, Y., Matsuoka, T., Kaneto, H., Watada, H., Fujitani, Y., Kishimoto, M., Sakamoto, K., Matsuhisa, M., Kawamori, R., Yamasaki, Y. and Hori, M. (1999). Induction of glycation suppresses glucokinase gene expression in HIT-T15 cells, *Diabetologia*, **42**, 1417–1424.

[29] MacDonald, M. J., Fahien, L. A., McKenzie, D. I. and Moran, S. M. (1990). Novel effects of insulin secretagogues on capacitation of insulin release and survival of cultured pancreatic islets, *Am. J. Physiol.*, **259**, E548–554.

